# Chemical and Nutritional Characterization of Seed Oil from *Cucurbita maxima* L. (var. Berrettina) Pumpkin

**DOI:** 10.3390/foods7030030

**Published:** 2018-03-01

**Authors:** Domenico Montesano, Francesca Blasi, Maria Stella Simonetti, Antonello Santini, Lina Cossignani

**Affiliations:** 1 Department of Pharmaceutical Sciences Section of Food Science and Nutrition, University of Perugia, Via San Costanzo, 06126 Perugia, Italy; domenico.montesano@unipg.it (D.M.); francesca.blasi@unipg.it (F.B.); maria.simonetti@unipg.it (M.S.S.); lina.cossignani@unipg.it (L.C.); 2 Department of Pharmacy, University of Napoli Federico II, via D. Montesano 49, 80131 Napoli, Italy

**Keywords:** pumpkin seed oil, fatty acids, stereospecific analysis, sterols, alcohols

## Abstract

Pumpkin (*Cucurbita* spp.) has received considerable attention in recent years because of the nutritional and health-protective value of seed oil. The nutritional composition of pumpkin native to central Italy, locally known as “Berrettina” (*Cucurbita maxima* L.), was evaluated. In particular, the lipid fraction of seed oil was characterized, and the triacylglycerol (TAG) was thoroughly studied by using a stereospecific procedure to obtain the intrapositional fatty acid composition of the three *sn*-positions of the glycerol backbone of TAG. Moreover, alkaline hydrolysis was carried out to study the main components of the unsaponifiable fraction, i.e., sterols and alcohols. It was observed that monounsaturated fatty acids and polyunsaturated fatty acids were the most abundant (41.7% and 37.2%, respectively) in Berrettina pumpkin seed oil, with high content of oleic and linoleic acid (41.4% and 37.0%, respectively). The main sterols of Berrettina pumpkin seed oil were Δ^7,22,25^-stigmastatrienol, Δ^7,25^-stigmastadienol, and spinasterol; with regard to the alcoholic fraction, triterpenic compounds were more abundant than aliphatic compounds (63.2% vs. 36.8%). The obtained data are useful to evaluate pumpkin seed oil from a nutritional point of view. The oil obtained from the seed could be used as a preservative and as a functional ingredient in different areas, e.g., cosmetics, foods, and nutraceuticals.

## 1. Introduction

The pumpkin (*Cucurbita* spp.), one of the most popular vegetables consumed in the world, has been recently recognized as a functional food [1,2,3]. Pumpkin seeds, generally considered agro-industrial waste, are an extraordinarily rich source of bioactive compounds with interesting nutraceutical properties [4]. In recent years, several studies [5,6,7] have highlighted the health properties of pumpkin seed oil against many diseases, including hypertension, diabetes, and cancer. It also shows antibacterial, antioxidant, and anti-inflammatory properties [8,9]. Due to the presence of interesting natural bioactive compounds, such as carotenoids, tocopherols, and sterols, pumpkin-derived products have a wide spectrum of biological activity, proven by in vivo experiments [10].

Because of the positive health effects, research has been focused particularly on the content and composition of fatty acids (FA) and tocopherols in pumpkin seed oil, while, to a lesser extent, other lipid components, such as sterols, alcohols, and phenol acids, have been studied, as is done with other food matrices to identify specific markers characteristic of the plant varieties [11]. Among the relevant aspects to be considered when dealing with this vegetable, the beneficial effects of using environmentally friendly natural herbicides [12] must be mentioned, since the content of bioactive compounds could be affected, and there could be possible contamination of this vegetable due to the presence of *Fusarium* spp. microfungi and their secondary metabolites [13], affecting the content of beneficial compounds of the vegetable itself. 

Stevenson et al. [14] summarized FA composition and reported significant differences among various cultivars of pumpkin seed oil extracted from various pumpkin sources. Rezig et al. [15] studied the chemical composition and oil properties of seeds of a Tunisian variety of pumpkin, Béjaoui (*C. maxima*). They found that the major FA were oleic, linoleic, and palmitic acids and that the seed oil was rich in δ-tocopherol, while the sterol marker was β-sitosterol and the predominant phenolic acid was syringic acid. Siano et al. [16] highlighted that saturated FA (SFA) and monounsaturated FA (MUFA) of *C. maxima* produced in southern Italy showed similar values (25.20% and 25.54%, respectively), while the polyunsaturated FA (PUFA) content was 48.14%. Habib et al. [17] determined the proximate composition of powdered seed and the lipid composition of the oil of *C. maxima* collected in Bangladesh. They affirmed that the high degree of unsaturation makes the oil suitable for use as valuable drying agent, and lower free FA content indicates suitability of the oil for consumption as food. 

Other researchers studied the chemical composition of pumpkin seed oils from *C. pepo* [5,18,19,20,21]. Due to the differences among the species and/or varieties of *Cucurbita* spp. grown in different areas of the world, the present study focused on characterizing a native Italian cultivar (*C. maxima*, var. Berrettina, locally known as “priest’s hat”), paying attention to the lipid composition of the seed oil. Since there is little information about the lipid structure, the present research deepens understanding of the total FA content and intrapositional composition of Berrettina pumpkin by using stereospecific analysis, and pays attention to other minor lipid components such as sterols, alcohols, and carotenoids. Butinar et al. [22] proposed high-performance liquid chromatography (HPLC) analysis of triacylglycerol (TAG) as a useful technique to evaluate the genuineness of pumpkin seed oils produced in Slovenia, but to the best of our knowledge, there are no data in the literature dealing with stereospecific analysis of pumpkin seed TAG.

## 2. Materials and Methods

### 2.1. Materials and Chemicals

Methanol (MeOH), diethyl ether, petroleum ether, formic acid, hydrochloric acid, and acetone were purchased from J.T. Baker B.V. (Deventer, the Netherlands). Hexane, ethanol (EtOH), chloroform (CHCl_3_), anhydrous sodium sulfate (Na_2_SO_4_), and potassium hydroxide (KOH) were bought from Carlo Erba Reagents (Milan, Italy). Deionized water (>18 MΩ cm resistivity) was obtained from a Milli-Q SP Reagent Water System (Millipore, Bedford, MA, USA). Supelco™ 37 Component FAME Mix, containing the methyl esters of 37 fatty acids (Supelco, Bellefonte, PA, USA; catalog No. 47885-U), was used. Lipase from porcine pancreas (EC 3.1.1.3), *sn*-1,2-diacylglycerol kinase from *Escherichia coli* (DAGK; EC 2.7.1.107), cholesterol (≥99%), ergosterol (≥95%), stigmasterol (~95%), β-sitosterol (≥95%), 5-α-cholestane (≥97%), γ-linolenic acid (≥99%), 2′,7′-dichlorofluorescein, *N*,*O*-bis(trimethylsilyl)trifluoroacetamide (BSTFA), 1-octadecanol (99%), 1-docosanol (98%), 1-octacosanol (≥99%), lutein (≥97%), and β-carotene (≥97%) were purchased from Sigma-Aldrich (St. Louis, MO, USA). 

### 2.2. Collection of Pumpkin Samples

Pumpkin belongs to the family *Cucurbitaceae*. The samples (*C. maxima* L., var. Berrettina) were taxonomically identified by Luigi Frassineti (Tuder Green Service, Todi, Italy). It is a leafy green vegetable with medium-large flattish fruits with green-gray, moderately hard knobby skin, edible yellow/orange flesh, and a central cavity with numerous plump, whitish-yellow seeds. Three pumpkins cultivated in central Italy (Todi) and collected in autumn 2016 were selected for their uniformity of shape, weight, and color. The fresh pumpkin samples were weighed (about 2.5 kg each), peeled, and, after manual removal of seeds, cut into small pieces (1.5 cm × 1.5 cm × 1.5 cm) and analyzed. The seeds were cleaned to remove impurities and dried at 60 °C for 24 h in a hot-air fan oven. After that, the seeds were reweighed until the weight was constant. The samples were stored in a dry place in the dark at room temperature.

### 2.3. Determination of Pumpkin Chemical Composition

Crude fat, protein, moisture, and ash contents of pumpkin samples were determined according to the procedures described in the Association of Official Analytical Chemists (AOAC) method [23]. 

### 2.4. Seed Lipid Extraction

Dried pumpkin seed samples were ground using a kitchen grinder (Oster, model 869-50R, Lakewood, CA, USA). Extraction of lipid fraction of the pumpkin seeds was performed using petroleum ether as a solvent in a Soxhlet extractor, according to AOAC procedure [23]. The extract was dried over Na_2_SO_4_, and then the solvent was evaporated under reduced pressure using a rotary evaporator (Büchi Rotavapor B-480, Essen, Germany) at 40 °C. Finally, the residue was weighed and dissolved in hexane. The recovered oil was stored at 4 °C until use.

### 2.5. Isolation of TAG Fraction from Oil Samples

The TAG fraction was isolated by thin layer chromatography (TLC), according to the method described by Cossignani et al. [24], from total fat of pumpkin seed samples using silica gel plates (SIL G-25, 0.25 mm, 20 cm × 20 cm; Macherey-Nagel, Germany) and petroleum ether/diethyl ether/formic acid (70:30:1, *v/v/v*) as a developing solvent. The TAG fraction was scraped off, extracted with hexane/diethyl ether (1:1, *v/v*), subjected to transesterification, and analyzed by high-resolution gas chromatography (HRGC) as reported in Section 2.6 to obtain the constituent fatty acid methyl esters (FAME). The obtained data represent the total composition of FA esterified in all 3 *sn*-positions of TAG, named A_t_.

#### 2.5.1. Stereospecific Analysis of TAG

The stereospecific analysis procedure [25] carried out on TAG of pumpkin seed oil isolated as reported in the previous paragraph involved the following steps:

- The pancreatic lipase procedure (Section 2.5.2) to obtain the FA% intrapositional composition of *sn*-2 position of glycerol backbone of TAG, named A_2_;
- Preparation of *sn*-1,3/*sn*-1,2(2,3)-diacylglycerol (DAG), followed by the DAGK enzymatic procedure (Section 2.5.3), to obtain the FA% intrapositional composition of *sn*-1 and *sn*-2 positions of glycerol backbone of TAG, named A_1,2_.

#### 2.5.2. Pancreatic Lipase Procedure

Hydrolysis of TAG was carried out according to the method provided by the Italian fat and derivate control standards (Norme Italiane per il Controllo dei Grassi e Derivati (NGD) method) [26]. Tris-HCl buffer (pH 8.08), bile salts, CaCl_2_, and pancreatic lipase were added to an aliquot of TAG. The mixture was incubated under magnetic stirring in a water bath at 40 °C for 5 min, and then 6 M HCl and diethyl ether were added and the mixture was centrifuged. Diethyl ether was dried by anhydrous Na_2_SO_4_ and evaporated under nitrogen flow to small volume. The hydrolytic products were separated on TLC plates, and the developing solvent system was petroleum ether/diethyl ether/formic acid (70:30:1, *v/v/v*). The band corresponding to *sn*-2-monoacylglycerols (*sn*-2-MAG), visualized with 2′,7′-dichlorofluorescein spray, was scraped off, methylated, and analyzed by HRGC as reported in Section 2.6 to obtain the constituent FAME. The obtained data represent the intrapositional composition of FA esterified in *sn*-2 position, named A_2_.

#### 2.5.3. *sn*-1,3/*sn*-1,2(2,3)-Diacylglycerol (DAG) Preparation

An aliquot of TAG was dissolved in anhydrous diethyl ether, and freshly prepared ethyl magnesium bromide in anhydrous diethyl ether was added. The mixture was shaken, and then pentane (0.1% acetic acid) and water were added. The solution was vortexed and centrifuged (ALC 4218 centrifuge, Thermo Scientific, Waltham, MA, USA). The water was removed and the organic phase was dried over anhydrous Na_2_SO_4_ and concentrated with solvent removal under nitrogen stream. The DAG mixture was applied to TLC plates previously treated with 5% boric acid solution (methanol/water, 80:20, *v/v*) and then activated for 1 h at 120 °C. The developing system was hexane/diethyl ether (60:40, *v/v*). The band containing the *sn*-1,2(2,3)-DAG fraction (Rf ≈ 0.30; Rf of the *sn*-1,3-DAG band ≈ 0.35), located by iodine vapor exposition, was scraped off and extracted with diethyl ether.

#### 2.5.4. DAGK Enzymatic Procedure

The *sn*-1,2(2,3)-DAG ethereal solution was concentrated under nitrogen stream, then cardiolipin solution, buffered *sn*-1,2-DAGK, buffer, and Na_2_ATP were added, mixing each time. After incubation at 40 °C for 90 min under constant stirring, chloroform/methanol (2:1, *v/v*) was added to the mixture to stop the reaction and extract the products of interest. The combined extracts were concentrated, treated with anhydrous Na_2_SO_4_, and applied to TLC plates. The developing system was chloroform/methanol/25% ammonia (65:25:5, *v/v/v*). The band of the *sn*-1,2-phosphatidic acids (*sn*-1,2-PA), visualized with 2′,7′-dichlorofluorescein spray (Rf ≈ 0.1), was scraped off, methylated, and analyzed by HRGC with flame ionization detection (FID) as reported in the following paragraph to obtain the constituent FAME. The obtained data represent the intrapositional composition of FA esterified in *sn*-1,2 positions, named A_1,2_.

The FA composition at the *sn*-1- and *sn*-3-positions was obtained applying the following formulas:

A_1_ = 2A_1,2_ − A_2_ (1)

A_3_ = 3A_t_ − A_2_ − A_1_ (2)

where A_1_ = % intrapositional composition of FA esterified in *sn*-1 position; A_1,2_ = % intrapositional composition of FA esterified in *sn*-1 and *sn*-2 positions; A_2_ = % intrapositional composition of FA esterified in *sn*-2 position; A_t_ = % total composition of FA esterified in all 3 *sn*-positions of TAG; A_3_ = % intrapositional composition of FA esterified in *sn*-3 position.

### 2.6. Preparation of FAME and HRGC-FID Analysis

The FAME of TAG, *sn*-2-MAG, and *sn*-1,2-PA fractions were prepared by transesterification. Hexane and 2 N methanolic KOH were added to the fraction and stirred for 3 min, then water was added. The organic phase (upper) containing the FAME was dried over anhydrous Na_2_SO_4_, then analyzed by HRGC. A DANI GC1000 DPC gas chromatograph (Norwalk, CT, USA) equipped with a split-splitless injector and a flame ionization detector (FID) was used. Separation was obtained using the CP-Select CB for FAME fused silica capillary column (50 m × 0.25 mm i.d., 0.25 μm f.t.; Varian, Superchrom, Milan, Italy). Chromatograms were acquired and processed using Clarity integration software (DataApex Ltd., Prague, Czech Republic). The injector and detector temperature was 250 °C. The oven temperature was held at 180 °C for 6 min, and raised to 250 °C at 3 °C/min; the final temperature was held for 10 min. Carrier gas (He) flow rate was 1 mL/min; the injection volume was 1 µL with a split ratio of 1:70. A standard solution containing 37 FAME was used to identify the individual FA. The percentage of each FA was calculated using the peak area of the samples. The data were normalized considering only the main reported FA (% mol mean values ≥0.1). 

### 2.7. Alkaline Hydrolysis of Pumpkin Seed Oil

Alkaline hydrolysis of pumpkin seed oil was carried out according to the method reported by Cossignani et al. [27]. Prior to alkaline hydrolysis, 0.2% 5-α-cholestane in CHCl_3_ and 1% 1-octacosanol (used as internal standards) were added to the oil samples, then the American Oil Chemists’ Society (AOCS) method (Ch 6–91) was used [28]. The products obtained after alkaline reaction were applied to TLC silica gel plates previously treated with 0.2 N KOH in MeOH, then activated for 1 h at 100 °C. The developing system was hexane/diethyl ether (65:35, *v/v*). The band containing the sterols, visualized with 2′,7′-dichlorofluorescein spray, was scraped off and extracted with CHCl_3_. Then the solvent was evaporated and removed under nitrogen stream.

### 2.8. Preparation of Trimethylsilyl Ether Derivatives and HRGC-FID Analysis

The silylation reaction was carried out as described by Lombardi et al. [29] with slight modifications. In brief, BSTFA and acetone were added to the sterol fraction and the reaction was carried out at 40 °C for 20 min to obtain trimethylsilyl ether (TMSE) derivatives. Sterols and alcohols, as TMSE derivatives, were analyzed using a DANI GC1000 DPC gas chromatograph equipped with a split-splitless injector and FID. 

Separation of TMSE sterols and alcohols was obtained using the AT-1701 fused silica capillary column (25 m × 0.25 mm i.d., 0.2 μm f.t.; Alltech, Milan, Italy). 

For analysis of TMSE sterols, the following chromatographic conditions were used: injector and detector temperature was 300 °C; oven temperature of 260 °C was held for 4 min, then increased to 300 °C at 1.5 °C/min, and the final temperature was held for 30 min; carrier gas (He) flow rate was 1.2 mL/min. 

For analysis of TMSE alcohols, the following chromatographic conditions were used: injector and detector temperature was 290 °C; oven temperature was held at 180 °C for 3 min, raised to 260 °C at 6 °C/min for 15 min, then raised to 280 °C at 2 °C/min for 30 min. 

Chromatograms were acquired and processed using Clarity integration software. The percentage of each sterol was calculated by using the peak area of the samples corrected with the correction factor equal to 1 as reported by Laakso [30]. TMSE sterols were also analyzed by HRGC coupled with mass spectrometry (MS) detector as described in Section 2.9.

### 2.9. HRGC-MS Analysis

A Shimadzu GCMS-QP2010 gas chromatograph equipped with a quadrupole mass spectrometer (Shimadzu, Milan, Italy) and split-splitless injector maintained at 300 °C was used. The following MS parameters were used: interface temperature 270 °C; MS ionization mode electron ionization; detector voltage 0.9 kV; acquisition mass range 50–500 u; scan speed 1000 u/s; acquisition mode full scan; scan interval 0.5 s; solvent delay 6 min. Data were collected by GC-MS Solution software (Shimadzu). The column and the chromatographic conditions were the same as those reported in Section 2.8. 

TMSE sterols and alcohols were identified by comparing retention times and mass spectra to those of authentic TMSE-derivatized compounds. Confirmation of these structures was achieved by HRGC-MS using the National Institute of Standards and Technology (NIST; Gaithersburg, MD, USA) 2008 library to match mass spectral peaks of phytosterol standards to those found in pumpkin seed oil samples. Comparisons of parent molecular ion (M+) and fragmentation ions/patterns were employed to assist in elucidating the identities of the phytosterols. In addition to the presence of specific ion fragments, the relative intensity of the ion fragments was considered. Some compounds, for which commercial standards are not available, were tentatively identified by comparison of relative retention times, M+ values, and fragmentation patterns with data obtained from olive oil analysis or from MS spectra reported in the literature. 

### 2.10. Carotenoid Analysis

The seed oil carotenoids were analyzed by HPLC with diode-array and mass spectrometry detection systems (DAD-MS). To perform this determination, a seed oil sample was diluted fourfold and injected into the HPLC system. Quali-quantitative determination of carotenoids was carried out using the HPLC-DAD-MS validated method described in Blasi et al. [31].

### 2.11. Statistical Analysis

FA, sterol, alcohol, and carotenoid composition data are reported as mean values and standard deviation (SD). HRGC analyses were carried out in duplicate. Data were processed and edited with Microsoft Excel 2016 (Microsoft, Redmond, MA, USA).

## 3. Results and Discussion

### 3.1. Nutritional Composition and Caloric Value

Data on nutritional composition and caloric value of pumpkin (*C. maxima*, var. Berrettina) are reported in Table 1. The samples had a high content of water (82.50%); in fact, a variety of vegetables have water composition in the range of 80–90%. The flesh is characterized by a low fat content. Simple sugars and ash showed similar values (0.82% and 0.84%, respectively), while the protein content was higher (1.28%). Data relative to moisture, ash, and protein are in good agreement with those reported by Kim et al. [32] for Korean pumpkin (*C. maxima*) flesh. Generally, the proximate composition is extremely variable [32,33,34], due to the differences among the species and/or varieties of *Cucurbita* spp. grown in different areas of the world. The low caloric value of pumpkin (*C. maxima*, var. Berrettina) is 25.35 kcal/100 g, according to data in the literature [35,36]. 

### 3.2. Fatty Acid Composition of Seed Oils and Nutritional Quality Index

Total FA% compositions, corresponding to each FA component of oil and TAG fraction, are reported in Table 2. SFA with carbon chains shorter than 14 carbon atoms, called short- and medium-chain FA, was not found in pumpkin seed oil, as confirmed in other papers [14,20,37,38]. SFA was represented especially by palmitic (C16:0) and stearic (C18:0) acids, at 14.2% and 5.8%, respectively. It is reported that oils rich in myristic (C14:0) and palmitic acids affect the ratio of total to high-density lipoprotein (HDL) cholesterol only a little, and stearic acid slightly reduces this ratio [35]. PUFA and MUFA fractions were the most abundant (37.2% and 41.7%, respectively, for oil; 37.8% and 43.0%, respectively, for TAG); in fact, the main FA were oleic (C18:1*n*-9) and linoleic (C18:2*n*-6) acids. Berrettina pumpkin seed oil showed a higher content of oleic acid than linoleic acid (41.4% vs. 37.0% for oil); on the contrary, Procida et al. [20] reported a higher content of linoleic acid (44.30–51.58%) than oleic acid (34.16–42.59%) for three Italian samples of pumpkin (Crudigno, Pepo, and Winter). It has been reported by some authors [14] that oleic acid is the predominant FA (41–46%), followed by linoleic acid (33.4–34.3%), in pumpkin seed oil from Italy and Libya. Siano et al. [16] found that the main FA of southern Italian pumpkin (*C. maxima*) seed oil were linoleic acid (47.45%), followed by oleic (25.54%) and palmitic (17.58%) acids. Habib et al. [17] found that pumpkin (*C. maxima*, known as “Misti Kumra”) seed oil contained a high amount of oleic acid, 40.58%, while linoleic acid was 14.97%.

It was reported by Orsavova et al. [38] that MUFA may reduce low-density lipoprotein (LDL) cholesterol, while it may possibly increase HDL cholesterol, and that oleic acid (C18:1*n*-9) may promote insulin resistance contrary to PUFA, with protection against insulin resistance. The high content of linoleic acid is an important nutritional aspect, because it is an essential FA (EFA), together with linolenic acid (C18:3*n*-3), and a lack of either of the two leads to ill health and causes deficiency symptoms. In addition, several studies [39] have positively correlated EFA intake with reduction of numerous disorders (cardiovascular, neurological, visual, and cancerous). 

Minor FA (contents lower than 0.5%) of Berrettina pumpkin seed oil were myristic, palmitoleic (C16:1*n*-7), margaric (C17:0), heptadecenoic (C17:1*n*-7), arachidic (C20:0), behenic (C22:0), and lignoceric (C24:0) acids. These data are in good agreement with similar studies [14,20,37,38].

The extreme variability of FA composition of pumpkin seeds, and consequently of the corresponding pumpkin seed oils, is affected not only by the variety of the cultivar, but also by the growth conditions and degree of ripeness [40]. 

In addition, the nutritional quality of pumpkin (*C. maxima,* var. Berrettina) cultivated in central Italy was evaluated, using different indices, based on the FA composition of the oils. It is known that some FA can help to prevent or promote coronary thrombosis and atherosclerosis based on their effects on LDL concentration and serum cholesterol [41]. The equations proposed by Ulbricht and Southgate [41] for the atherogenic index (AI) and thrombogenic index (TI) showed that C12:0, C14:0, and C16:0 FA are atherogenic, while C14:0, C16:0, and C18:0 are thrombogenic. PUFA *n*-3, PUFA *n*-6, and MUFA are antiatherogenic and antithrombogenic. Atherogenic indices have been described as powerful indicators of the risk of cardiovascular disease; the higher the value, the higher the risk of developing the disease, and vice versa. The AI of pumpkin (*C. maxima*) seed oil was lower than that reported by Siano et al. [16] (0.19 for Berrettina vs. 0.34), while the TI was comparable (0.50 for Berrettina vs. 0.65).

### 3.3. Stereospecific Analysis Data

The indirect method of analyzing TAG was based on a chemical–enzymatic–instrumental (stereospecific analysis) procedure and allowed us to carry out qualitative and quantitative analysis of all molecular TAG species, including enantiomeric ones. In fact, it is known that in TAG molecules the positions esterified by FA are numbered relative to their stereospecific numbering (*sn*) as *sn*-1, *sn*-2, and *sn*-3. The procedure allowed us to evaluate the FA % composition of each of the three *sn*-positions of TAG (% intrapositional composition). These data could be used to obtain the distribution of FA among the three *sn*-positions of TAG [24,25,42,43].

**Figure 1:**
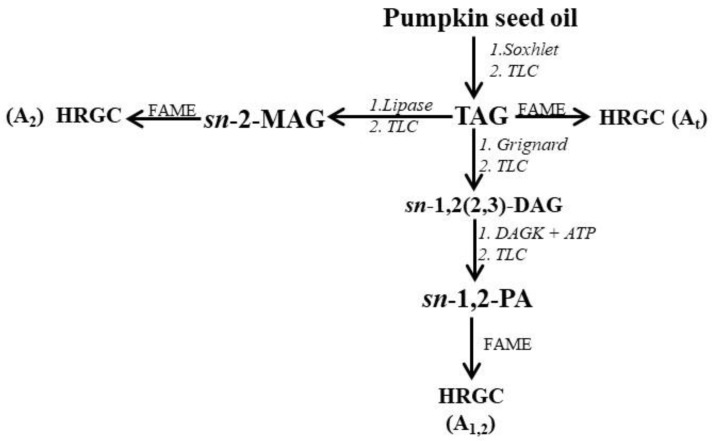
Scheme of the procedure used for stereospecific analysis of TAG from pumpkin (*C. maxima*, var. Berrettina) seed oil. HRGC: high-resolution gas chromatography; FAME: fatty acid methyl esters; TAG: triacylglycerol; *sn*-2-MAG: *sn*-2-monoacylglycerols; PA: phosphatidic acids; TLC: thin layer chromatography; DAG: diacylglycerol; DAGK: diacylglycerol kinase.

**Figure 2:**
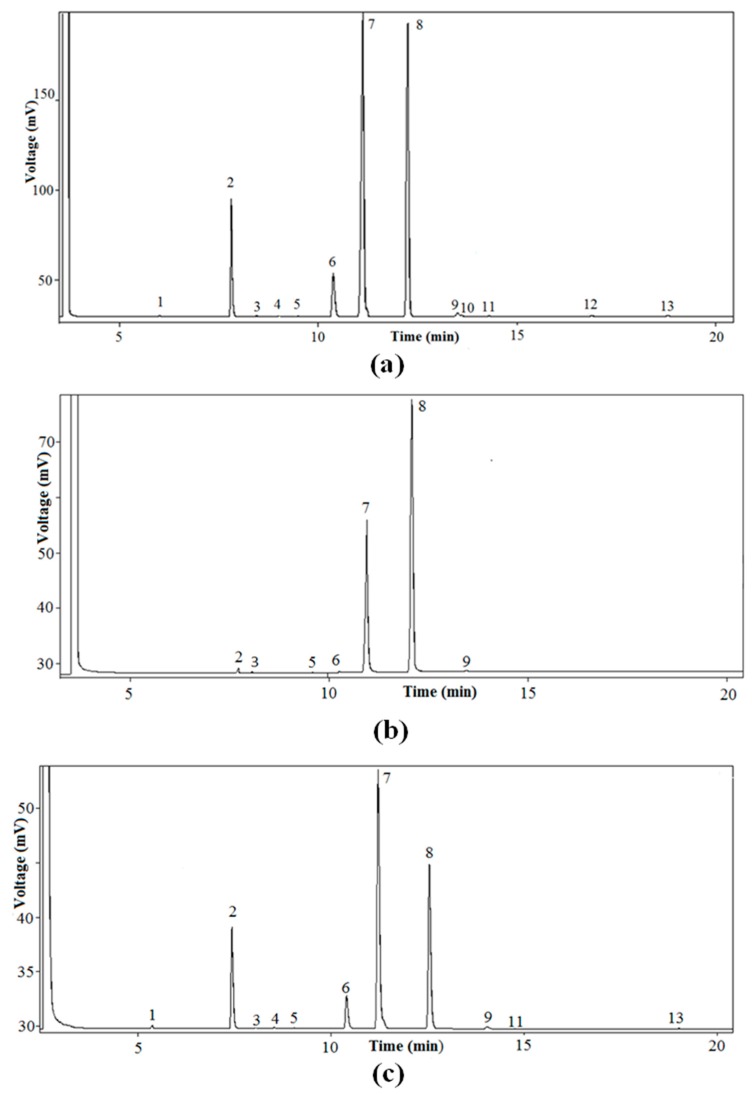
Characteristic high-resolution gas chromatography (HRGC) profiles of pumpkin (*C. maxima*, var. Berrettina) seed oil samples. (**a**) Fatty acid methyl esters (FAME) of TAG fraction; (**b**) FAME of monoacylglycerol (MAG) fraction; (**c**) FAME of PA fraction. 1. Myristic acid, 2. Palmitic acid, 3. Palmitoleic acid, 4. Margaric acid, 5. Heptadecenoic acid, 6. Stearic acid, 7. Oleic acid, 8. Linoleic acid, 9. Linolenic acid, 10. Arachidic acid, 11. Behenic acid, 12. Eicosenoic acid, 13. Lignoceric acid.

Initially, the lipid fraction was isolated by Soxhlet extraction, and then the TAG fraction was purified by TLC. As shown in Figure 1, several steps were carried out. Initially, the total FA% composition (A_t_) was determined by HRGC. Figure 2a shows the characteristic HRGC profile of the FAME of the TAG fraction of pumpkin samples. Then, enzymatic hydrolysis of TAG with pancreatic lipase was used to obtain *sn*-2-MAG, and finally, after HRGC analysis of the FAME (Figure 2b), the acidic composition of *sn*-2-position (A_2_) of the glycerol backbone of TAG was obtained. TAG was also subjected to chemical hydrolysis with Grignard reagent, then separation of enantiomeric *sn*-1,2(2,3)-DAG, realized by enzymatic synthesis of *sn*-1,2-PA, allowed us to obtain the acidic composition of the *sn*-1,2-positions (A_1,2_) of the glycerol backbone of TAG, after HRGC analysis of the FAME (Figure 2c). 

The stereospecific analysis represents a potent analytical-investigative procedure to give the fingerprint of TAG fraction for each botanical variety or animal species. The results of the stereospecific analysis procedure are shown in Table 2. It was observed that Berrettina pumpkin seed oil had a high percentage of UFA (98.5%) in *sn*-2 position, represented by MUFA (36.2%) and PUFA (62.4%). In *sn*-2 position, the main FA was linoleic acid (62.1%), followed by oleic acid (36.0%). SFA are preferentially esterified in *sn*-1 position (44.7%), represented essentially by palmitic and stearic acids (28.6% and 13.7%, respectively). Regarding the two primary positions, oleic acid was equally distributed between the *sn*-1 and *sn*-3 positions, while linoleic acid prefers the *sn*-3 position (42.2%). 

### 3.4. Unsaponifiable Fraction

Another part of the research was analyzing the main components of unsaponifiable fractions, i.e., sterols and alcohols. Alkaline hydrolysis was carried out on pumpkin seed oils to obtain data relative to the qualitative composition of sterol and alcohol fractions. Phytosterols have been studied for their role in lowering cholesterol levels. In addition to this property, plant sterols have antiatherogenic, anti-inflammatory, anticancer, and antioxidation activities [44]. Together with the high content of linoleic acid, sterols can help in the treatment of lipid-associated disorders such as atherosclerosis. 

In contrast to the other vegetable oils with Δ^5^-sterols (β-sitosterol, campesterol, and stigmasterol) as the major components, Wenzl et al. [45] showed that pumpkin seed oil contains specific Δ^7^-phytosterols, typical of only a few plant families (e.g., *Cucurbitaceae*), that provide a fingerprint for detection of adulteration. These Δ^7^-sterols are supposed to give the pumpkin seed oil a beneficial effect in the treatment and prophylaxis of disorders of the prostate gland and the urinary bladder [46]. 

In this paper, sterol identification was carried out by HRGC-MS. Each peak was analyzed via detection of the parent molecular ion and the fragmentation pattern of the TMSE derivative. In addition to the presence of specific ion fragments, the relative intensity of the ion fragments was considered. Some TMSE sterols were identified by comparison with the NIST mass spectra library; typical fragmentation is reported in Table 3, together with sterol composition (% and mg/100 g). TMSE sterols give a molecular ion that is not abundant, while the first significant ion observed in the high mass range was usually equivalent to [M-15]^+^, due to the loss of the methyl terminal group. Other main fragment ions useful for identifying the single sterol compounds are [M-90]^+^, [M-105]^+^, and [M-129]^+^. They correspond to the loss of the trimethylsilanol, methyl group with trimethylsilanol, and fragmentation of the 1,2-cyclopenthanophenanthrene structure, respectively. The predominant sterols of Berrettina pumpkin seed oil are Δ^7^-sterols, in particular Δ^7,22,25^-stigmastatrienol, Δ^7,25^-stigmastadienol, and spinasterol, which accounted for about 76.8% of the total sterols, followed by Δ^7^-avenasterol and Δ^7^-stigmastenol. The Δ^5^-sterols were represented by campesterol, stigmasterol, and only a little cholesterol, as in other foodstuff [47]. Differences between the contents of Δ^5^- and Δ^7^-sterols could be attributed to the maturity stage of seeds or to the solvent used in the extraction procedure [15]. A total sterol content of 295 mg/100 g oil was measured in *C. maxima* seed oil; 15.7 mg/100 g was represented by Δ^5^-sterols and 279.3 mg/100 g by Δ^7^-sterols. The total content was in agreement with other studies [9,48], even if a wide range of variability is reported [9]. Hence, more detailed examinations of the composition of the sterol fraction of this oil could be of special interest. For example, analysis of a more extensive sampling is required for better characterization of pumpkin seed oils and for their authentication. 

The alcoholic fraction (aliphatic and triterpenic classes) was also studied, after derivatization as TMSE and analysis by HRGC-MS; the typical fragmentation is reported in Table 4, together with the alcohol composition (% and mg/100 g). Some aliphatic alcohols (from C16 to C25 members of the 1-alkanol homologous series) with odd and even numbers of carbon atoms of the aliphatic chain were identified. The TMSE alcohols were identified by comparison with the NIST mass spectra library. TMSE alcohols give a molecular ion that is not abundant, while the first significant ion observed in the high mass range was equivalent to [M-15]^+^, due to the loss of the methyl terminal group, and [M-117]^+^, equivalent to the loss of [(CH3)_3_-Si-O]^+^, i.e., the OTMSi group. Moreover, HRGC-MS analysis of alcohols after BSTFA derivatization resulted in various peaks with MS fragments *m/z* 73, 75, 103, and 117 characteristics for OTMSi groups. The peak at *m*/*z* 129, corresponding to [(CH3)_3_-Si-O^+^=CH-CH=CH_2_]^+^, has been identified as the fragment originating from the breakdown of ring A along with the TMS moiety. Four main triterpenic alcohols were identified: butyrospermol, obtusifoliol, β-amyrine, and cycloartenol. The key fragmentation ions were molecular ion [M]^+^, [M-15]^+^, [M-90]^+^, and [M-105]^+^. Aliphatic alcohol content was 36.8%, of which hexadecanol and octadecanol were the most abundant (each about 4.4 mg/100 g oil), while triterpenic alcohol content was around 63.2%, of which obtusifoliol was the most abundant (11.9 mg/100 g oil). 

These minor compounds are also important constituents of edible oils and could be useful in distinguishing different pumpkin oil varieties.

### 3.5. Carotenoid Analysis

HPLC-DAD-MS analysis, performed directly on seed oils, detected two important carotenoids: lutein and β-carotene. Quantitative analysis showed a lutein concentration of 8 mg/L seed oil, while β-carotene concentration was 2.5 mg/L seed oil. 

Carotenoids such as lycopene, lutein, β-carotene, and zeaxanthin have been studied by many researchers both analytically and biologically [49,50,51]. Carotenoids represent a significant added value for food: various health properties have been associated with these compounds, including antioxidant and anticancer activity, photoprotection, protection against cardiovascular diseases, and anti-inflammatory activity [52,53,54].

## 4. Conclusions

The results reported in this study confirm that pumpkin seed oils are interesting vegetable oils with important nutritional value, related to the presence of MUFA, PUFA, phytosterols, and carotenoids. A more extensive sampling for a better characterization of pumpkin seed oils and for authentication purposes is necessary. To the best of our knowledge, this is the first time stereospecific analysis data of pumpkin seed oil have been reported. Information obtained from this research could help to assess the potential of seed oil from this pumpkin cultivar to be commercially exploited for nutraceutical application, and incorporated into food formulations to benefit human health. 

## Figures and Tables

**Table 1:** Nutritional composition (g/100 g edible part) of pumpkin (*C. maxima*, var. Berrettina) (mean value ± standard deviation (SD), *n* = 3).

Component	Mean Value ± SD
Energy (kcal/100 g)	25.35
Moisture	82.50 ± 0.37
Dry matter	17.50 ± 0.14
Total ash	0.84 ± 0.04
Crude protein	1.28 ± 0.03
Crude oil	0.08 ± 0.01
Total sugars	4.90 ± 0.09
Starch	4.10 ± 0.05
Simple sugars	0.82 ± 0.03

**Table 2:** Total and intrapositional % fatty acid composition (% mol, mean value ± SD, *n* = 3) of oil and triacylglycerol (TAG) fraction of pumpkin (*C. maxima*, var. Berrettina) seed oil.

		Total Lipids	TAG	*sn*-1-	*sn*-2-	*sn*-3-
Yield (%)		29.0 ± 0.9				
Saturated fatty acids (SFA)						
Mystiric acid	C14:0	0.2 ± 0.0	0.1 ± 0.0	0.5 ± 0.0	–	–
Palmitic acid	C16:0	14.2 ± 0.4	12.2 ± 0.4	28.5 ± 0.0	1.0 ± 0.0	7.1 ± 0.0
Margaric acid	C17:0	0.2 ± 0.0	0.1 ± 0.0	0.5 ± 0.0	–	–
Stearic acid	C18:0	5.8 ± 0.2	6.2 ± 0.2	13.7 ± 0.0	0.4 ± 0.0	4.4 ± 0.0
Arachidic acid	C20:0	0.5 ± 0.0	0.4 ± 0.0	–	–	1.1 ± 0.0
Behenic acid	C22:0	0.1 ± 0.0	0.2 ± 0.0	1.5 ± 0.0	–	–
Lignoceric acid	C24:0	0.1± 0.0	–	–	–	–
Total SFA		21.1	19.2	44.7	1.5	11.6
Monounsaturated fatty acids (MUFA)						
Palmitoleic acid	C16:1*n*-7	0.2 ± 0.0	0.2 ± 0.0	–	0.1 ± 0.0	0.4 ± 0.0
Heptadecenoic acid	C17:1*n*-7	0.1 ± 0.0	0.1 ± 0.0	–	0.1 ± 0.0	0.3 ± 0.0
Oleic acid	C18:1*n*-9	41.4 ± 0.7	42.7 ± 0.7	46.5 ± 0.0	36.0 ± 0.0	44.6 ± 0.0
Eicosenoic acid	C20:1*n*-9	0.1 ± 0.0	–	–	–	–
Total MUFA		41.7	43.0	46.4	36.2	46.3
Polyunsaturated fatty acids (PUFA)						
Linoleic acid	C18:2*n*-6	37.0 ± 0.5	37.4 ± 0.5	7.7 ± 0.0	62.1 ± 0.0	42.0 ± 0.0
Linolenic acid	C18:3*n*-3	0.2 ± 0.0	0.4 ± 0.0	1.1 ± 0.0	0.3± 0.0	0.1 ± 0.0
Total PUFA		37.2	37.8	8.8	62.4	24.1
Index						
UFA/SFA		3.7	4.2	1.2	65.7	6.1
MUFA/SFA		2.0	2.2	1.0	24.1	4.0
PUFA/SFA		1.8	2.0	0.2	41.6	2.1
PUFA*n*-6/PUFA*n*-3		185	93.5	7.0	207	420
UI		116.3	118.9	65.2	161.3	129.6
AI		0.19	0.16	0.55	0.01	0.10
TI		0.50	0.44	1.38	0.03	0.32

–: <0.1%; UI: unsaturation index: Σ(mol % of each FA) × (number of double bonds of each FA); AI: atherogenic index (C12:0 + 4 × C14:0 + C16:0)/(MUFA + PUFA*n*-6 + PUFA*n*-3); TI: thrombogenic index (C14:0 + C16:0 + C18:0)/(0.5 × MUFA + 0.5 × PUFA*n*-6 + 3 × PUFA*n*-3 + (PUFA *n*-3/PUFA*n*-6)).

**Table 3:** Fragmentation ions used for identification of trimethylsilyl ether (TMSE) sterols of pumpkin (*C. maxima*, var. Berrettina) seed oil and sterol composition (% and mg/100 g oil; mean value ± SD, *n* = 3).

Sterol	[M]^+^	[M-15]^+^	[M-90]^+^	[M-105]^+^	[M-129]^+^	%	mg/100 g
Cholesterol	458	443	368	353	329	0.3 ± 0.1	0.9 ± 0.1
Campesterol	472	457	382	367	343	3.3 ± 1.0	10.3 ± 1.0
Stigmasterol	484	469	394	379	355	1.6 ± 0.4	4.5 ± 0.8
Spinasterol	484	469	394	379	–	20.5 ± 1.9	61.8 ± 4.2
Δ^7,25^-Stigmastadienol	484	469	394	379	–	26.4 ± 2.3	78.4 ± 5.5
Δ^7,22,25^-Stigmastatrienol	482	467	392	377	–	29.9 ± 2.5	91.2 ± 6.8
Δ^7^-Stigmastenol	486	471	396	381	–	5.7 ± 1.0	15.1 ± 1.5
Δ^7^-Avenasterol	484	469	394	379	–	12.3 ± 0.9	32.8 ± 2.8
Δ^5^-Sterol (total)						5.2	15.7
Δ^7^-Sterol (total)						94.8	279.3

[M]^+^ indicates molecular ion. [M-15]^+^, [M-90]^+^, [M-105]^+^, and [M-129]^+^ correspond to loss of the methyl group, trimethylsilanol, methyl group with trimethylsilanol, and to fragmentation of the 1,2-cyclopenthanophenanthrene structure, respectively.

**Table 4:** Fragmentation ions used for identification of TMSE alcohols of pumpkin (*C. maxima*, var. Berrettina) seed oil and alcohol composition (% and mg/100 g oil; average value ± SD, *n* = 3).

**Aliphatic Alcohols**	**[M]^+^**	**[M-15]^+^**	**[M-90]^+^**	**[M-117]^+^**	**%**	**mg/100 g**
Hexadecanol	314	299	224	195	7.4 ± 2.0	4.3 ± 1.0
Heptadecanol	328	313	238	209	3.1 ± 0.9	1.9 ± 0.3
Octadecanol	342	327	252	223	7.5 ± 1.3	4.4 ± 0.9
Nonadecanol	356	341	266	237	3.4 ± 0.8	2.9 ± 1.5
Eicosanol	370	355	280	251	4.9 ± 1.0	2.0 ± 0.8
Docosanol	398	383	308	279	3.1 ± 0.9	2.2 ± 0.3
Tricosanol	412	397	322	293	1.8 ± 0.5	1.3 ± 0.3
Tetracosanol	426	411	336	307	3.6 ± 0.5	1.2 ± 0.2
Pentacosanol	440	425	350	321	2.3 ± 0.7	1.5 ± 0.9
Total					36.8	21.7
**Triterpenic Alcohols**	**[M]^+^**	**[M-15]^+^**	**[M-90]^+^**	**[M-105]^+^**		
Butyrospermol	498	483	408	393	13.7 ± 1.2	8.1 ± 2.1
Obtusifoliol	484	469	394	379	20.2 ± 1.5	11.9 ± 1.1
β-Amyrine	498	483	408	393	16.1 ± 1.1	9.5 ± 1.8
Cycloartenol	498	483	408	393	13.3 ± 1.0	7.8 ± 1.5
Total					63.2	37.3

[M]^+^ indicates molecular ion. For aliphatic alcohols: [M-15]^+^, [M-90]^+^, and [M-117]^+^ correspond to loss of the methyl group [-CH_3_], trimethylsilanol [-OSi(CH_3_)_3_], and ethyl trimethylsilanol [-CH_3_CH_2_-OSi(CH_3_)_3_]. For triterpenic alcohols: [M-15]^+^, [M-90]^+^, and [M-105]^+^ correspond to loss of the methyl group [-CH_3_], trimethylsilanol [-OSi(CH_3_)_3_], and methyl group with trimethylsilanol [-CH_3_OSi(CH_3_)_3_].
